# RNA-seq reveals co-dysregulated circular RNAs in the adenomyosis eutopic endometrium and endometrial–myometrial interface

**DOI:** 10.1186/s12905-022-01871-2

**Published:** 2022-07-15

**Authors:** Zhengchen Guo, Hua Duan, Sha Wang, Sirui Wang, Qi Lin, Yazhu Li

**Affiliations:** Department of Minimally Invasive Gynecology, Beijing Obstetrics and Gynecology Hospital, Capital Medical University, Beijing Maternal and Child Health Care Hospital, No. 17 Qihelou Street, Dongcheng District, Beijing, 100006 China

**Keywords:** Adenomyosis, circRNA, RNA sequencing, Expression profile, Endometrial–myometrial interface, Endometrium

## Abstract

**Background:**

Uterine adenomyosis is associated with chronic pelvic pain, abnormal uterine bleeding, and infertility. The pathogenesis of adenomyosis is still unclear. Circular RNAs (circRNAs) have been implicated in several benign diseases and malignant tumors. We aimed to explore the co-dysregulated circular RNA profile in the eutopic endometrium and endometrial–myometrial interface (EMI) of adenomyosis.

**Methods:**

Total RNA was extracted from the eutopic endometrium and EMI of 5 patients with adenomyosis and 3 patients without adenomyosis. Next-generation sequencing was performed to identify the circRNA expression profile of the two tissue types. Bioinformatics analysis was performed to predict circRNA-binding miRNAs and miRNA-binding mRNAs and construct ceRNA networks, and functional enrichment analysis was performed to predict the biological functions of circRNAs.

**Results:**

Among the adenomyosis patients, 760 circRNAs were significantly upregulated and 119 circRNAs were significantly downregulated in the EMI of adenomyosis, while 47 circRNAs were significantly upregulated and 17 circRNAs were significantly downregulated in the eutopic endometrium of adenomyosis. We identified hsa_circ_0002144 and hsa_circ_0005806 as co-upregulated and hsa_circ_0079536 and hsa_circ_0024766 as co-downregulated in the eutopic endometrium and EMI. Bioinformatics analysis was performed to construct a ceRNA network of codifferentially expressed circRNAs. The MAPK signaling pathway is the most important signaling pathway involved in the function of the ceRNA network.

**Conclusions:**

Co-dysregulated circRNAs were present in the eutopic endometrium and EMI of adenomyosis. MiRNA binding sites were observed for all of these circRNAs and found to regulate gene expression. Co-dysregulated circRNAs may induce the eutopic endometrial invagination process through the MAPK signaling pathway and promote the progression of adenomyosis.

## Background

Adenomyosis is an estrogen-dependent benign uterine disease characterized by the presence of heterotopic endometrial glands and stroma in the myometrium with a depth of at least 2.5 mm at the time of histological diagnosis and reactive fibrosis of the surrounding smooth muscle cells of the myometrium [1, 2]. Moreover, this disease is associated with different types of symptoms, including cyclical uterine pain or chronic pelvic pain, dyspareunia, abnormal uterine bleeding such as menorrhagia, spotting or bleeding before and after menses, infertility, and even asymptomatic symptoms [3]. Therefore, the diagnosis of adenomyosis has long troubled clinicians, and coexisting endometriosis and leiomyomas further complicates the clinical diagnosis [4, 5]. Although improvements in imaging techniques, such as transvaginal ultrasonography and magnetic resonance imaging, have somewhat facilitated its diagnosis, histology after laparoscopic or laparotomy hysterectomy remains the ultimate diagnosis method and the gold standard treatment for women who have no desire for future fertility because of the lack of drug therapy specific for adenomyosis [5, 6]. Complex treatment and diagnostic difficulties underscore the importance of understanding the pathogenesis and progression of adenomyosis.

Although the exact pathogenic mechanisms are still poorly understood, the most widely accepted theory is the invagination of endometrial basalis into the myometrium upon tissue injury and repair (TIAR) [7]. According to the theory of invagination, the local hyperestrogenic environment of the uterus leads to abnormal contraction of the myometrium and subsequent trauma to the endometrial–myometrial interface (EMI), which is also known as the uterine junction zone [8]. The EMI, defined as the inner 1/3 of myometrium between endometrium and myometrium, is a highly specialized uterine compartment that is hormone-dependent and has different structural and biological characteristics with respect to the outer myometrium [9–11]. It plays a vital role in sperm transport, embryo implantation, placentation, and menstrual shedding [12]. In addition to structural abnormalities due to recurrent TIAR, the EMI in patients with adenomyosis presents aberrant expression of receptor molecules and signaling pathway regulation [13, 14]. Our team identified that the oxytocin receptor was expressed at higher levels in the EMI of adenomyosis than in normal myometrium [15]. The Lin28B/Let-7a axis has been suggested to be involved in the pathogenesis of adenomyosis by promoting the proliferation of EMI smooth myometrial cells [16].

The eutopic endometrium adjacent to the EMI is another important uterine compartment involved in the theory of invagination. It has been demonstrated that eutopic endometrial cells have enhanced motility and proliferative capacity to invade the myometrium through a disrupted EMI, thus causing adenomyosis [17, 18]. P21-activated kinase 1 in the eutopic endometrium of adenomyosis, which is closely related to invasiveness, has been shown to be overactivated compared to normal endometrium [19]. Additionally, Nan et al. demonstrated that miR-124-3p regulates the migration and epithelial–mesenchymal transition of endometrial stromal cells by downregulating neuropilin 1 [20]. However, whether a common or interactive mechanism occurs in both the eutopic endometrium and EMI to promote metastasis and myometrial invasion of the endometrium has not been revealed.

Circular RNAs (circRNAs) are covalently closed, endogenous biomolecules with tissue-specific and cell-specific expression patterns in eukaryotes, and they perform important biological functions by acting as microRNA or protein inhibitors (also known as ceRNA), regulating protein function or translating themselves [21]. Furthermore, circRNAs have been implicated in diseases such as diabetes mellitus, neurological disorders, cardiovascular diseases and cancer [22–25]. For instance, Botai et al. provided evidence that circNDUFB2 is involved in the degradation of IGF2BPs and activation of antitumor immunity during the progression of non-small-cell lung cancer by regulating protein ubiquitination and degradation as well as cellular immune responses [26]. Although several studies have been performed on circRNAs in gynecologic malignancies, few studies have focused on the role of circRNAs in benign gynecologic diseases [27, 28]. Our team conducted a preliminary exploration of the pathogenesis of circRNA in adenomyosis and found that enhanced circPVT1 may be involved in the pathogenesis of adenomyosis by stimulating endometrial cell proliferation and invasion [29].

To comprehensively investigate the mechanism of circRNAs in uterine adenomyosis, the present study first performed circRNA sequencing and a differential expression analysis to explore the expression profile of circRNAs in the EMI and eutopic endometrium of adenomyosis. Then, the target miRNAs and mRNAs for common differentially expressed circRNAs (DECs) in both sites were predicted to establish a ceRNA network; and finally, a functional enrichment analysis was performed to identify the main roles played by circRNAs in uterine adenomyosis.

## Methods

### Sample collection

Sixteen samples of the eutopic endometrium and EMI from patients receiving hysterectomy diagnosed with and without adenomyosis (eutopic endometrium and EMI were paired sample and from one patient) pathologically between August 2020 and March 2021 were collected in the Department of Minimally Invasive Gynecology, Beijing Obstetrics and Gynecology Hospital, Capital Medical University. Normal endometrium and EMI were obtained from 3 patients in control group who receiving total hysterectomy for cervical cancer, two patients with stage IA1 and one patient with stage IB1. Eutopic endometrium and EMI were obtained from 5 patients with adenomyosis in study group. Endometrium and EMI were collected during the operation. The diagnosis of adenomyosis was confirmed by histological examination by two pathologists as the presence of ectopic endometrial tissue (endometrial stroma and glands) within the myometrium [30]. The exclusion criteria included postmenopausal or perimenopausal status; administration of preoperative hormone therapy or gonadotropin-releasing hormone agonist (GnRHa) suppression for at least three months before tissue sampling; diagnosis of endometrial disease, such as endometrial carcinoma and atypical endometrial hyperplasia; diagnosis of pelvic inflammatory disease; and diagnosis of chronic diseases, such as coronary heart disease and hypertension. All participants were fully informed and provided signed informed consent in accordance with the Declaration of Helsinki. The study received approval from the Beijing Obstetrics and Gynecology Hospital, Capital Medical University Ethics Committee. Tissues were stored in liquid nitrogen for testing.

### Total RNA isolation and quality control

Total RNA was extracted from tissues using the TRIzol reagent (12183-555, Invitrogen, Carlsbad, CA, USA) according to the manufacturer’s instructions. For tissue samples, grind about 50 mg with liquid nitrogen into powder and transfer the powder samples into the 2 ml tube contains 1.5 ml Trizol reagent. The mix was centrifuge at 12,000×*g* for 5 min at 4 °C. The supernatant was transferred to a new 2.0 ml tube which was added 0.3 ml of Chloroform/isoamyl alcohol (24:1) per 1.5 ml of Trizol reagent. After the mix was centrifuged at 12,000×*g* for 10 min at 4 °C, the aqueous phase was transferred to a new 1.5 ml tube which was add equal volume of supernatant of isopropyl alcohol. The mix was centrifuged at 12,000×*g* for 20 min at 4 °C and then removed the supernatant. After washed with 1 ml 75% ethanol, the RNA pellet was airdried in the biosafety cabinet and then dissolved by add 200 µL of DEPC-treated water. Subsequently, total RNA was quantified by measuring the absorbance at 260 nm and the purity at 260/280 and 260/230 nm ratios, using a NanoDrop system(Thermo Fisher Scientific, MA, USA), to exclude the presence of proteins, phenol and other contaminants. RNA integrity was measured using the RNA integrity number (RIN) value by Agilent 2100 bioanalyzer(Thermo Fisher Scientific, MA, USA), which was calculated based on the comparison of the areas of 18S rRNA and 28S rRNA[31]. RIN over a threshold of 7.0 were considered to indicate non-degraded RNA extraction methods.

### CircRNA library construction and sequencing

Total RNA was treated with DNase I, which degrades double-stranded and single-stranded DNA present in RNA samples. Ribosomal RNA was removed using the Ribo-off rRNA Depletion Kit (Vazyme, Inc.). Linear RNA was removed using RNase R (Epicenter, lnc.). Purification was performed using Agencourt RNAClean XP magnetic beads. The circular RNA molecules were fragmented and purified. Immediately following the RNA cleanup, first-strand cDNA synthesis was performed using directional RT buffer, followed by second-strand synthesis using the directional second strand buffer. End repaired and Y-Adapter ligation to A-tailed cDNA was followed by PCR. The amplified product was dissolved in Elution Buffer, and the library construction was completed. The library was qualified and quantitated by two methods: the distribution of the fragment size was checked using an Agilent 2100 bioanalyzer and the library was quantified using Qubit Fluorometer(Thermo Fisher Scientific, MA, USA). Finally, the qualified libraries were paired-end sequenced on MGISEQ-2000 (BGI-Shenzhen, China) platform with 100 bp read length.

### Alignment and prediction

We downloaded the reference genome (hg19) and gene model annotation files directly from the genome website. Bwa (version 0.7.17-r1188) and bowtie2 (version 2.2.8) were used to build the reference genome index and align the paired-end clean reads to the reference genome [32, 33]. CircRNA predictions were performed using CIRI2 (version 2.0.6) and find_circ (version 1.2) software separately and intersected based on the location in the chromosome [34, 35].

### Differential expression analysis

For the circRNA expression analysis, each count was normalized using junction reads per billion mapped reads (RPB). Normalized read count = 10^9^ C/S, where C is the read count of a circRNA and S is the sum of the circRNA read count in a sample. A differential expression analysis was performed with the DESeq2 R package (version 1.30.0) between adenomyosis and normal samples. CircRNAs that were only upregulated or downregulated in the eutopic endometrium and EMI were filtered with the thresholds of |log_2_Fold change|> 0.5 and 8, respectively, and an adjusted *p* value < 0.05.

### Establishment of the circRNA–miRNA-mRNA network

According to the hypothesis that circRNA could indirectly regulate mRNA expression by competing with miRNA as a natural sponge in the cytoplasm, the ceRNA network was constructed using the following steps: (1) miRanda v3.3a software for linux system (https://anaconda.org/bioconda/miranda/3.3a/download/osx-64/miranda-3.3a-hb4d813b_3.tar.bz2) and the circBank database (downloaded into R) were used to predict miRNA binding sites of circRNA [36]; (2) starBase (https://starbase.sysu.edu.cn/) and TargetScan were used to forecast the target genes of the miRNAs and build the miRNA–mRNA interaction pairs [37]; (3) circRNA–miRNA pairs were integrated with miRNA–mRNA pairs to build a circRNA–miRNA–mRNA triple regulatory network, which was visualized using Cytoscape 3.8.2 software (https://cytoscape.org/).

### Function enrichment analyses

To gain further insights into the molecular mechanisms and functions of the aberrantly expressed circRNAs, Gene Ontology (GO) and Kyoto Encyclopedia of Genes & Genomes (KEGG) enrichment analyses were carried out using the Database for Annotation, Visualization and Integrated Discovery (DAVID, v6.8) [38]. The top ten GO terms and the top 20 enriched KEGG pathways were visualized by R. Significant results were defined with a *p* < 0.05.

### Signaling pathway-related ceRNA network

The mitogen-activated protein kinase (MAPK) signaling pathway-related ceRNA network was selected based on the target genes involved in the most enriched KEGG pathway, and it was visualized using Cytoscape software. We presented the target genes in a general diagram of the MAPK signaling pathway using DAVID.

## Results

### Identification of differentially expressed circRNAs in tissues with adenomyosis

**Fig. 1:**
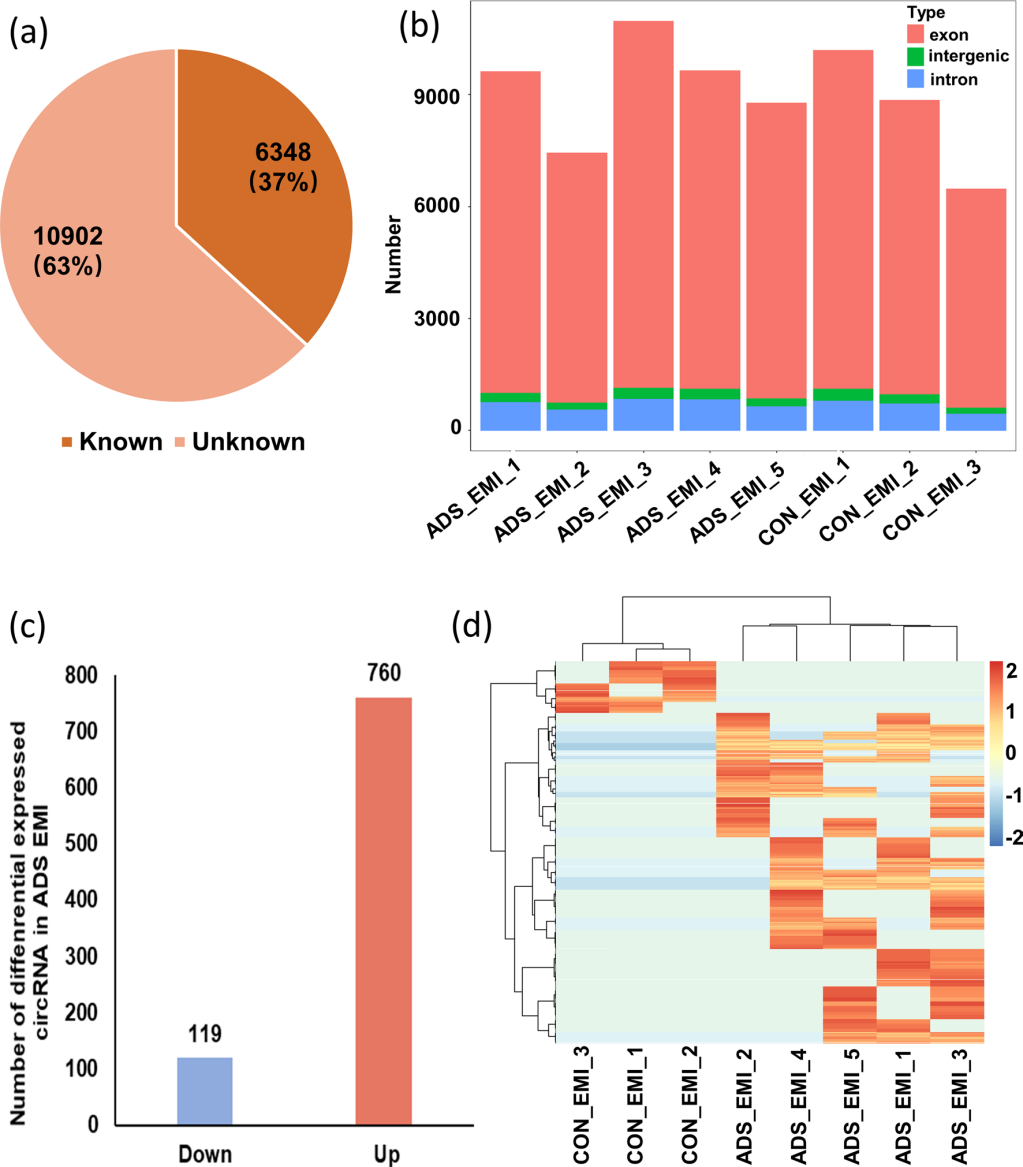
Characteristics of the circular RNA expression profile in EMI tissues from patients of adenomyosis and control group. **a** The identified circRNAs in this study were compared with previously reported circRNAs in the circBase database. **b** Genomic origin of the identified circRNAs. **c** Box plots showing the distribution of differential expressed circRNAs. **d** Hierarchical clustering of the significantly differentially expressed circRNAs. ‘Red’ indicates higher expression, and ‘Blue’ indicates lower expression. ADS, adenomyosis; CON, control, EMI, endometrium–myometrium interface; circRNA, Circular RNA

We compared the expression pattern of circRNAs between the adenomyosis EMI and control EMI from eight patients. A total of 17,250 distinct circRNA candidates were detected between the adenomyosis EMI and control EMI. We compared the detected circRNAs with the circBase database [39] and found that 6348 (37%) circRNAs were previously reported and 10,902 (63%) circRNAs were newly identified in our study (Fig. 1a). The detected circRNAs were primarily derived from protein-coding exons. A small proportion of the circRNAs were derived from introns and intergenic regions of the genome (Fig. 1b). We next analyzed the different expression levels of known circRNAs in the EMI between patients with adenomyosis and the control group. Only circRNAs upregulated or downregulated in five adenomyotic eutopic endometria were filtered (|log_2_Fold change|> 8, adjusted *p* value < 0.05). Compared with the control EMI, 119 circRNAs were significantly downregulated and 760 circRNAs were significantly upregulated, and they were all derived from protein-coding genes (Fig. 1c). Hierarchical clustering showed distinguishable circRNA expression patterns between the EMI with adenomyosis and control groups (Fig. 1d).

**Fig. 2:**
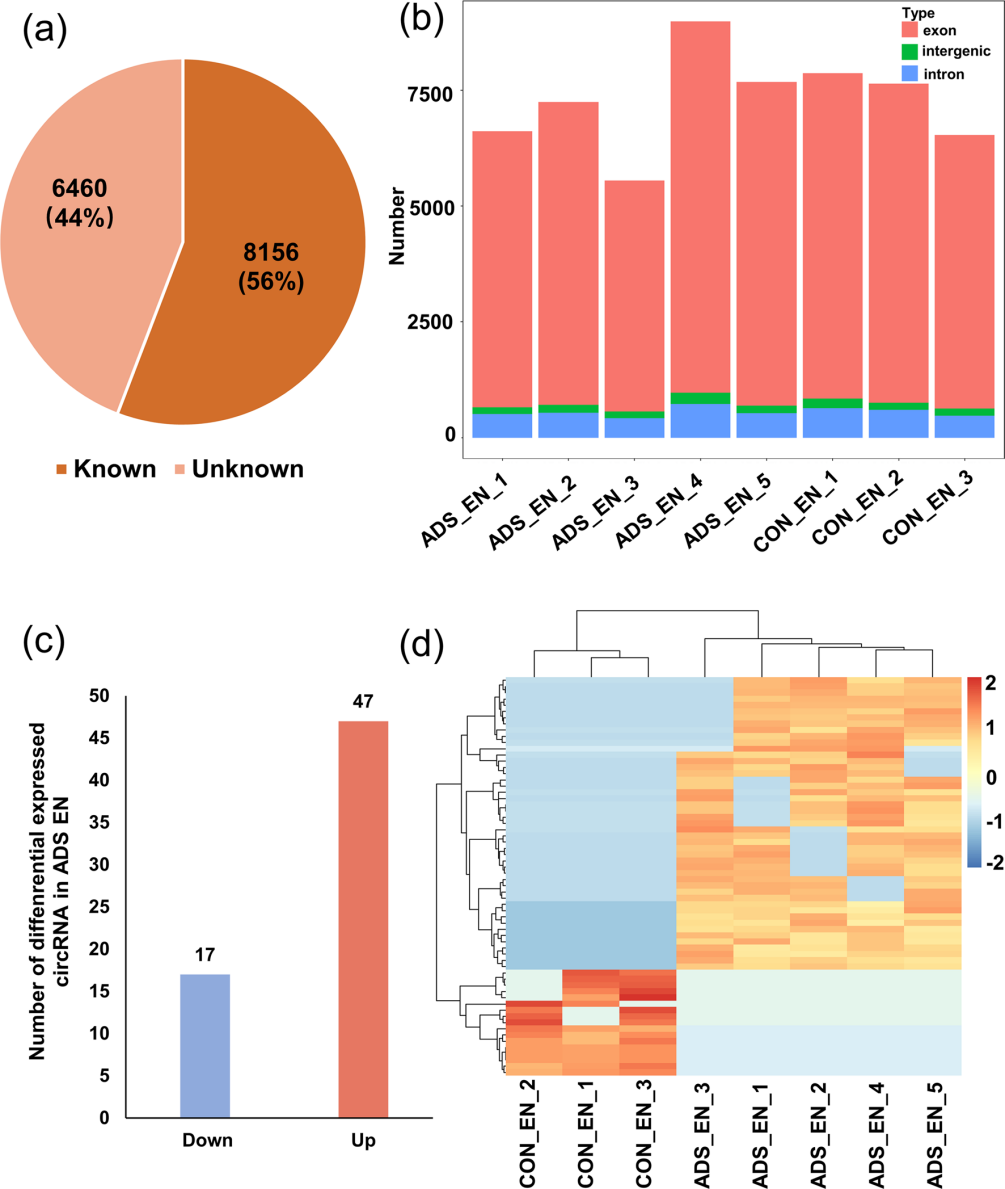
Characteristics of the circular RNA expression profile in eutopic endometrium tissues from patients with adenomyosis and control group. **a** The identified circRNAs in this study were compared with previously reported circRNAs in the circBase database. **b** Genomic origin of the identified circRNAs. **c** Box plots showing the distribution of differential expressed circRNAs. **d** Hierarchical clustering of the significantly differentially expressed circRNAs. ‘Red’ indicates higher expression, and ‘Blue’ indicates lower expression. ADS, adenomyosis; CON, control; EN, eutopic endometrium; circRNA, Circular RNA

We compared the expression pattern of circRNAs between adenomyotic eutopic endometrium and control endometrium from eight patients. A total of 14,616 distinct circRNA candidates were detected in both the adenomyotic eutopic endometrium and control endometrium. We compared the detected circRNAs with circBase databases and found that 8156 (56%) circRNAs were previously reported and 6460 (44%) circRNAs were newly identified in our study (Fig. 2a). The detected circRNAs were mainly derived from protein-coding exons. A small proportion of the circRNAs were derived from introns and intergenic regions of the genome (Fig. 2b). We next analyzed the different expression levels of known circRNAs in adenomyosis eutopic endometrium and control endometrium. Only circRNAs upregulated or downregulated in five adenomyotic eutopic endometria were filtered (|log_2_Fold change|> 0.5, adjusted *p* value < 0.05). Compared with the control endometrium, 17 circRNAs were significantly downregulated and 47 circRNAs were significantly upregulated in the adenomyotic eutopic endometrium, and they were all derived from protein-coding genes (Fig. 2c). Hierarchical clustering showed distinguishable circRNA expression patterns between the adenomyotic eutopic endometrium and control endometrium (Fig. 2d).

### Establishment of codysregulated circRNA-associated ceRNA networks in adenomyosis

**Fig. 3:**
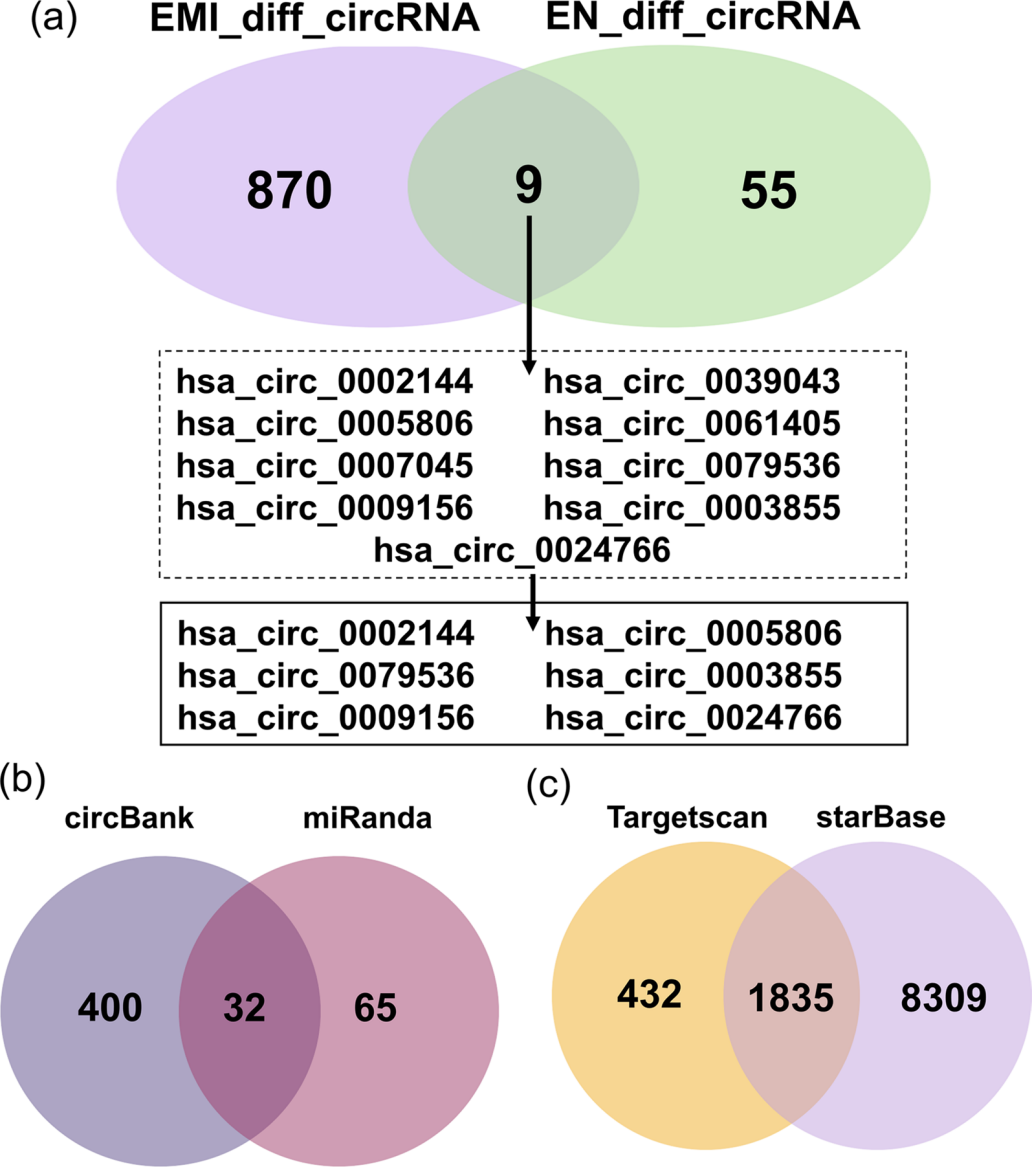
Co-dysregulated circRNAs and prediction of targeted RNA in eutopic endometrium and EMI. **a** Co-dysregulated circRNAs in eutopic endometrium and EMI. **b** Targeted miRNAs of co-dysregulated circRNAs. **c** Targeted mRNAs of co-dysregulated circRNAs targeted miRNAs. EMI_diff_circRNA, differential expressed circular RNAs in endometrium–myometrium interface; EN_diff_circRNA, differential expressed circular RNAs in eutopic endometrium

According to the previous hypothesis, there may be common biological processes of eutopic endometrium and EMI that induce eutopic endometrium to cross EMI, leading to uterine adenomyosis. First, we obtained nine circRNAs that were differentially expressed in both tissues from the same patient. Then, we screened six circRNAs with consistent expression trends, including three co-upregulated circRNAs (hsa_circ_0002144, hsa_circ_0005806, hsa_circ_0009156) and three co-downregulated circRNAs (hsa_circ_0079536, hsa_circ_0003855, hsa_circ_0024766). These circRNAs will be subjected to subsequent interactions for prediction (Fig. 3a).

**Fig. 4:**
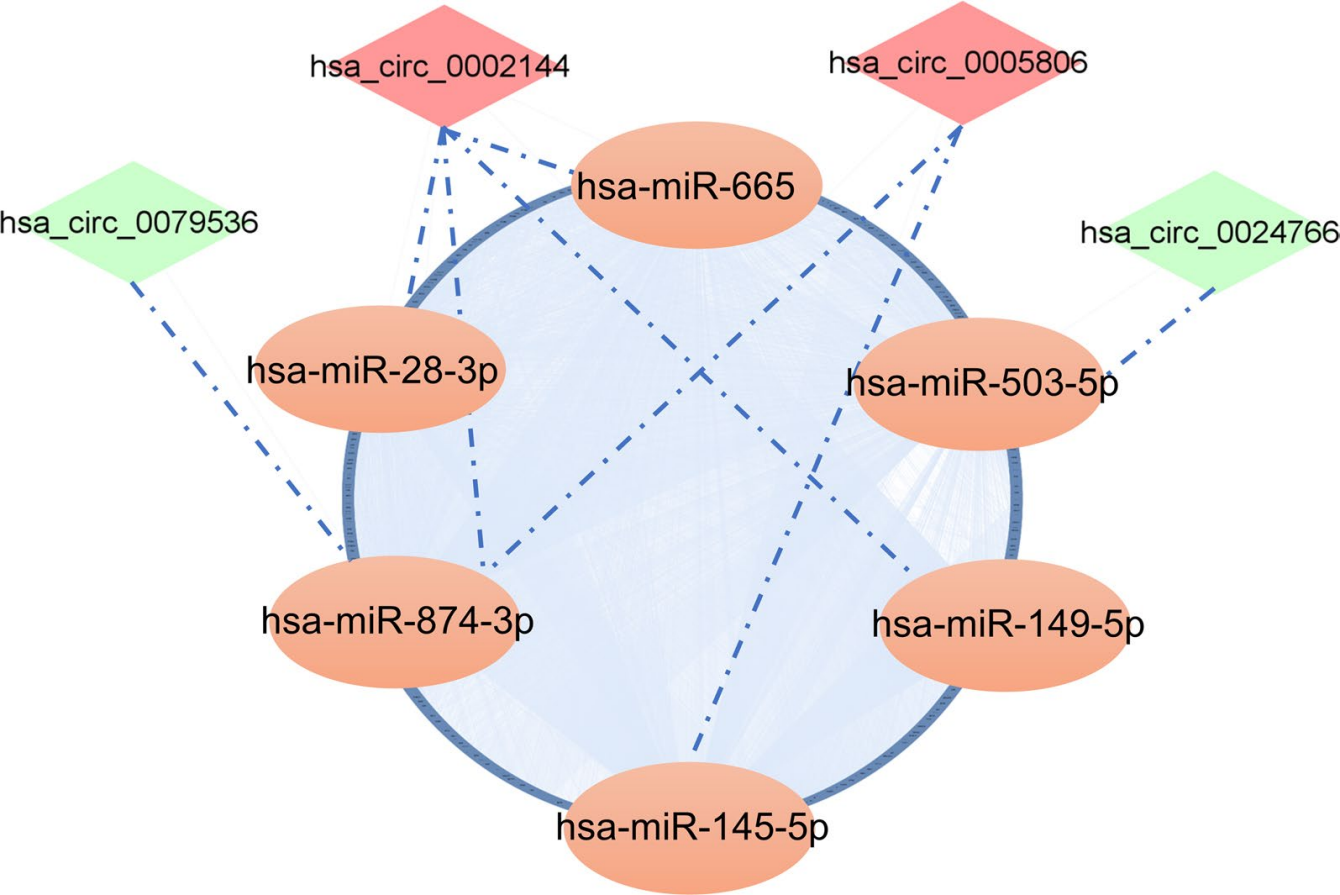
CeRNA network of co-dysregulated circRNAs. The mapping network included the 4 circRNAs with significant expression differences in the circRNA–miRNA network prediction. The blue ring consists of 1775 rounded rectangles representing the targeted genes

Numerous studies have been conducted to prove that circRNAs regulate gene expression by sponging miRNAs and blocking their biological functions [21, 40]. To determine the potential functions of codifferentially expressed circRNAs, circRNA–miRNA interactions were predicted by conserved seed-pairing sequence analysis using the miRanda and circBank databases. A total of 32 target miRNAs were selected from the common predicted circRNAs and are shown in the network (Figs. 3b, 4). MiRNA–mRNA interactions were predicted by searching for the presence of conserved 8-mer, 7-mer, and 6-mer sites that match the seed region of each miRNA using the TargetScan and starBase databases. A total of 1835 target mRNAs were selected from the common predicted mRNA and are shown in the network (Figs. 3c, 4). Ultimately, a ceRNA network of 4 circRNAs (hsa_circ_0002144, hsa_circ_0005806, hsa_circ_0079536, hsa_circ_0024766), 6 miRNAs and 1775 mRNAs was constructed by integrating the above circRNA–miRNA pairs and miRNA–mRNA pairs, and it was visualized by Cytoscape (Fig. 4).

### Functional enrichment analysis of coexpressed ceRNA

**Fig. 5:**
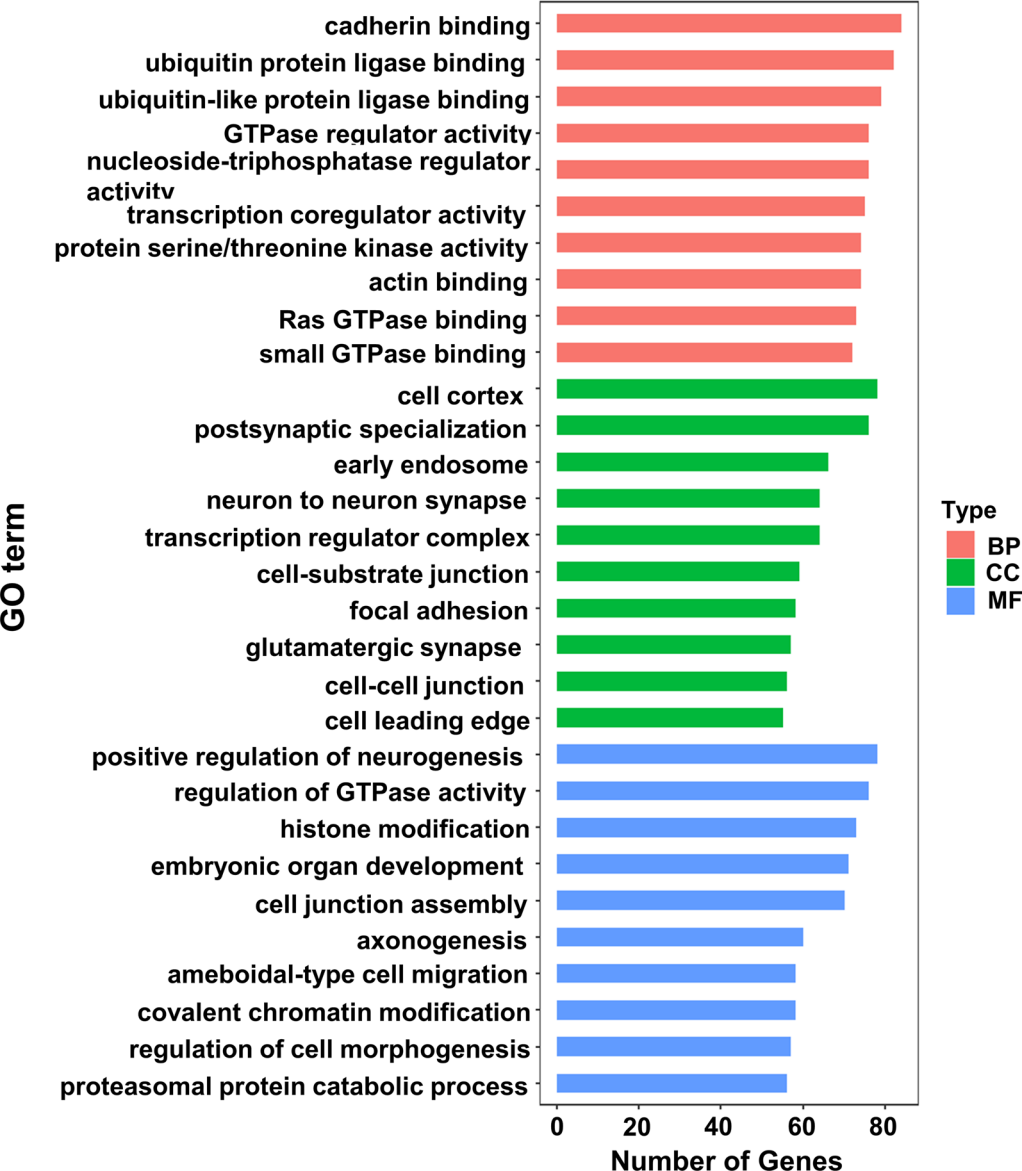
GO analysis of the potential targets of co-dysregulated circRNAs. GO, Gene ontology; BP, biological process; CC, cellular component; MF, molecular function

**Fig. 6:**
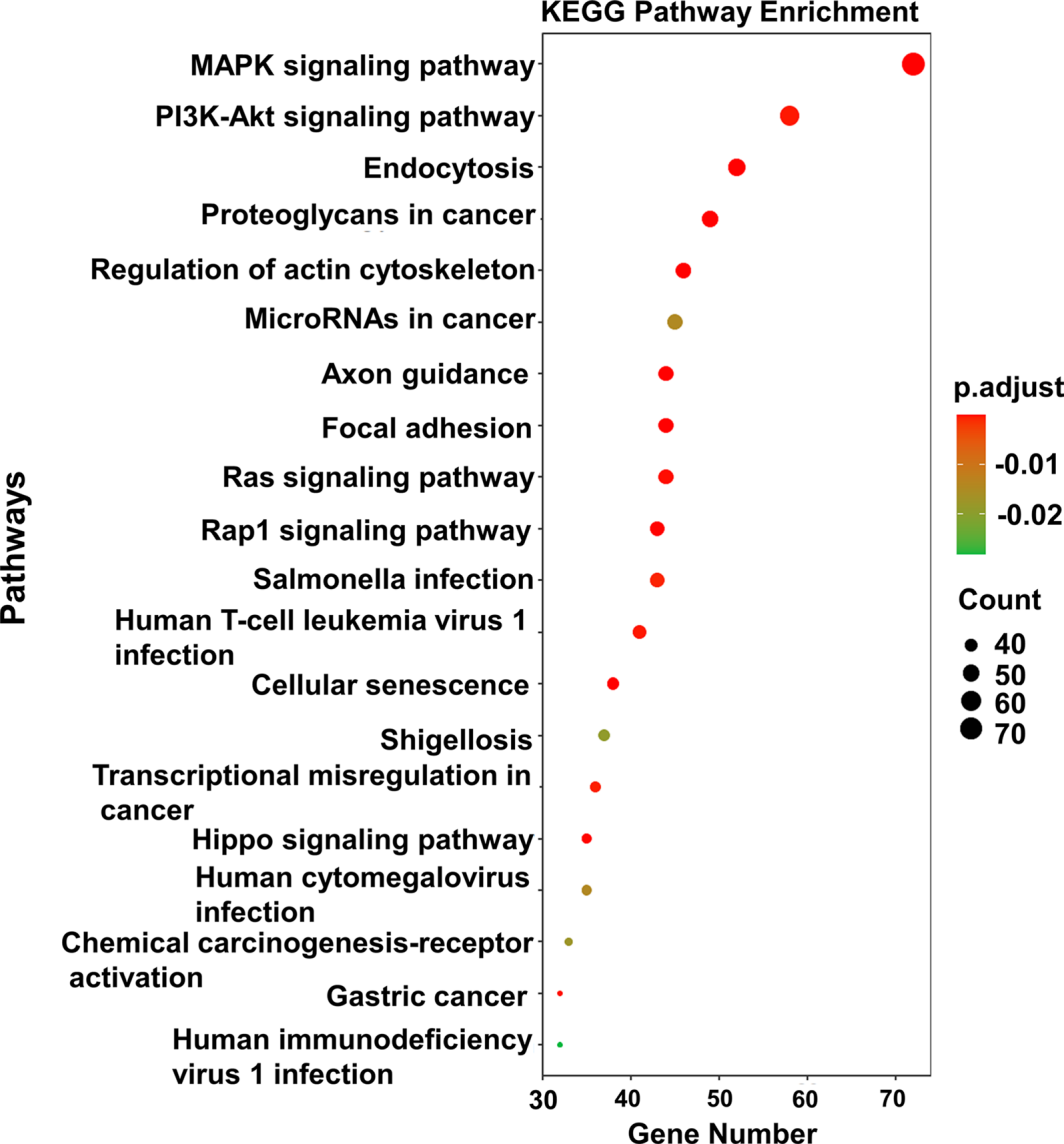
KEGG pathway analysis of the potential targets of co-dysregulated. The Bulb map shows the top 20 dysregulated KEGG pathways. KEGG, Kyoto Encyclopedia of Genes and Genome

To investigate the biological functions of the codysregulated circRNAs, we performed GO and KEGG pathway analyses with their target genes. The GO analysis results showed that the top two enriched GO terms for the biological process category were regulation of cadherin binding and regulation of ubiquitin protein ligase binding; the top two enriched terms for the cellular component category were cell cortex and postsynaptic specialization; and the top two terms for the molecular function category were positive regulation of neurogenesis and regulation of GTPase activity (Fig. 5). The KEGG pathway analysis indicated that the selected circRNAs mainly affect the MAPK signaling pathway. Other pathways, including phosphatidylinositol 3-kinase (PI3K)-Akt signaling, Ras, Hippo signaling and proteoglycans in cancer pathways, were also enriched in the KEGG analysis (Fig. 6).

### Identification of the MAPK signaling pathway-related ceRNA network

**Fig. 7:**
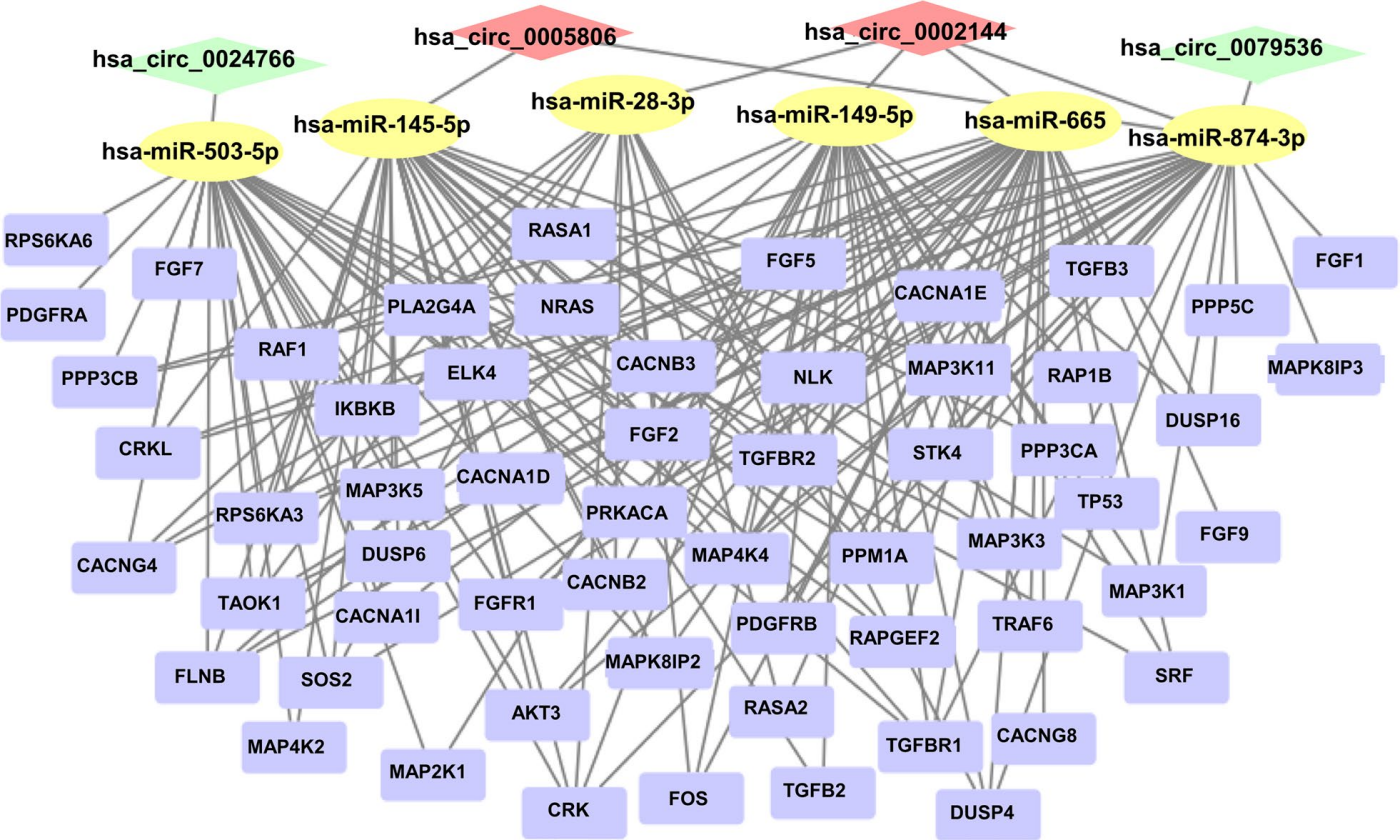
The mapping network related with MAPK signaling pathway

**Fig. 8:**
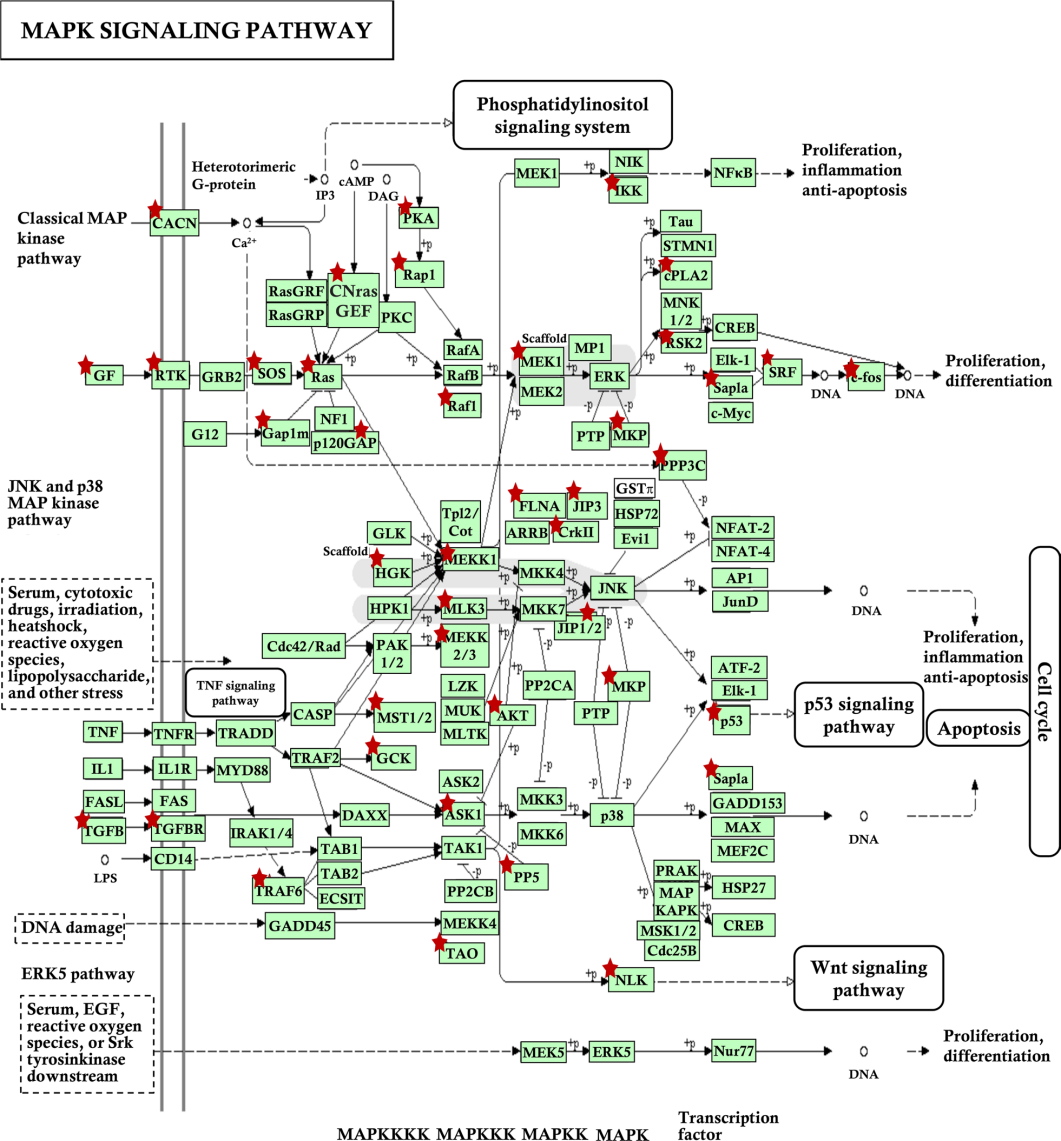
Targeted genes of network in schematic diagram of the MAPK signaling pathway. Red star denotes targeted gene

To further characterize the biological functions of eutopic endometrial and EMI co-dysregulated circRNAs in uterine adenomyosis, we extracted the MAPK signaling pathway-related circRNA interaction network from the ceRNA network (Fig. 7). Targeted genes are presented in the schematic diagram of the MAPK signaling pathway (hsa04010) from the KEGG pathway database (https://www.genome.jp/) (Fig. 8). Related genes are extensively involved in the classical MAPK pathway, c-Jun N-terminal kinase (JNK) and p38 MAPK pathway and extracellular signal-regulated kinase 5 (ERK5) pathway, affecting cell proliferation, differentiation, apoptosis, inflammation and other biological processes.

## Discussion

The pathophysiological process of uterine adenomyosis includes enhanced proliferation and migration of eutopic endometrial cells, along with abnormal contraction of EMI smooth muscle leading to tissue microtrauma that triggers TIAR, invasion of endometrial cells into the myometrium through EMI and colonization growth, and the occurrence of TIAR also enhances the activity of EMI smooth muscle cells. CircRNAs are critically involved in tumor cell proliferation, apoptosis, migration, and invasion [41, 42]. Upregulation of circRNA_100782 is suggested to significantly reduce the proliferation, migration and invasion of gastric cancer cells [43]. Overexpressed circRNA WHSC1 has been shown to promote the proliferation, migration and invasion of endometrial cancer cells and decrease apoptosis [44]. However, fewer studies have focused on circRNAs associated with adenomyosis. Several circRNAs have been reported to play regulatory roles in the pathogenesis of endometriosis, which is very similar to adenomyosis both in terms of pathogenesis and clinical manifestation. Hsa_circ_0008433 knockdown inhibited endometrial stromal cell proliferation, migration, colony formation, and angiopoiesis and promoted cell apoptosis in endometriosis [45]. While, Yang et al. revealed that knockdown of circ_0000673 promoted endometrial cell proliferation and migration [46]. Some of the biological processes in adenomyosis are similar to those in cancer and endometriosis, such as increased proliferative activity and invasive capacity and reduced apoptosis. Therefore, we hypothesize that circRNAs also play an essential role in adenomyosis.

As previously mentioned, there may be common abnormal molecular biological processes in the EMI and endometrium leading to uterine adenomyosis. Our study identified the upregulated circRNAs hsa_circ_0002144 and hsa_circ_0005806 and downregulated circRNAs hsa_circ_0079536 and hsa_circ_0024766 through an analysis of high-throughput sequencing data. No relevant studies of these circRNAs were found through a PubMed search; therefore, we further performed a bioinformatics analysis to predict circRNA-associated miRNAs and mRNAs and construct ceRNA networks to predict their biological functions. In this study, the GO analysis showed that the targeted genes in the ceRNA network were mainly involved in important biological processes, including cell adhesion and migration, protein hydrolysis, signal transduction and DNA transcription regulation. These biological processes illustrate the altered proliferation and invasive capacity of both eutopic endometrial cells and EMI smooth muscle cells in adenomyosis. Meanwhile, targeted genes in the network were involved in various important signaling pathways, such as the MAPK signaling pathway, PI3K-Akt signaling pathway, Ras signaling pathway and Hippo signaling pathway, according to the KEGG analysis, which have also been shown to be involved in adenomyosis [47–50]. The results suggest that eutopic endometrium and EMI smooth muscle co-expression of circRNAs alters cellular activity and influences the development of uterine adenomyosis by regulating these important signaling pathways. In addition, the expression of these genes is also involved in neurogenic and excitatory processes, suggesting that circRNA may be an important regulator of the dysmenorrheal in adenomyosis.

Our results indicate that circRNA regulates adenomyosis eutopic endometrium and EMI tissue activity mainly by regulating MAPK signaling pathway. The MAPK signaling pathway is revealed to plays an important role in complex cellular processes, such as proliferation, differentiation, development, transformation, and apoptosis [51]. Several studies have confirmed that circRNAs can regulate MAPK signaling pathway-related proteins through ceRNA action to exert corresponding effects [52–54]. Gao et al. provided the first evidence of the circ_0006528/miR-7-5p/Raf1/MEK/ERK regulatory network in the development of breast cancer [55]. Wang et al. revealed that the MAPK signaling pathway is involved in ovarian endometriosis through a functional analysis of circRNAs associated with this disease [56]. It has been proven in studies an implication of the MAPK/ERK pathways in proliferation of uterine smooth muscle cells from women with adenomyosis, and MAPK signaling pathway activation in adenomyosis eutopic endometrium [47, 57]. Therefore, we presumed that circRNA may affect the MAPK signaling pathway in the pathogenesis of adenomyosis, and our study validated this hypothesis.

This study has strengths and limitations. One of the strengths of this study is circRNA sequencing using next-generation sequencing technology was performed to comprehensively analyze differential expression of circRNAs in tissues from the patients with adenomyosis and their possible biological functions. The occurrence and development of adenomyosis does not result from alterations in one types of tissue alone. Therefore, another advantage of this study is that circRNAs co-expressed in eutopic endometrial and EMI tissues were identified and their functions were initially explored, based on the theory of endometrial invagination, to investigate the combined effects from the common alterations of both tissues on the development of uterine adenomyosis.

There are also limitations in this study. Firstly, there were fewer included cases in this study. Secondly, the expression profile of circRNA in this study was not experimentally validated in a larger sample of tissues and further experiments are needed to verify the function and mechanism of the ceRNA network in adenomyosis. In addition, our current study did not address ectopic lesions of adenomyosis, which have been considered to be many different from the eutopic endometrium. We will further explore the function of circRNAs in ectopic lesions and their possible correlation with eutopic endometrium and EMI in adenomyosis.

## Conclusions

We revealed the circRNA expression profiles associated with the eutopic endometrium and EMI and identified four codysregulated circRNAs in the two types of adenomyosis tissues. We also predicted the potential circRNA–miRNA–mRNA network and functions of the codifferentially expressed circRNAs. Notably, the MAPK signaling pathway was the most important signaling pathway involved in the function of the ceRNA network. This study may provide new genetic information for exploring the role of dysregulated circRNAs in adenomyosis as well as potential diagnostic biomarkers and therapeutic targets for adenomyosis.

## Data Availability

The datasets generated and/or analysed during the current study are available in the SRA database, PRJNA848212.
